# The role of borderline personality disorder symptoms on absenteeism & work performance in the Netherlands Study of Depression and Anxiety (NESDA)

**DOI:** 10.1186/s12888-020-02815-6

**Published:** 2020-08-24

**Authors:** Trees T. Juurlink, Femke Lamers, Hein J. F. van Marle, Johannes R. Anema, Aartjan T. F. Beekman

**Affiliations:** 1 Amsterdam UMC, Vrije Universiteit, Psychiatry, Amsterdam Public Health research institute, De Boelelaan 1117, Amsterdam, The Netherlands & GGZ inGeest Specialized Mental Health Care, Oldenaller 1, Amsterdam, The Netherlands; 2 Amsterdam UMC, Vrije Universiteit, Social Medicine, Amsterdam Public Health research institute, De Boelelaan 1117, Amsterdam, The Netherlands

**Keywords:** Borderline personality disorder, Depressive disorder, Anxiety disorder, Work performance, Work absence

## Abstract

**Background:**

Symptoms of borderline personality disorder (BPD) were previously found to be associated with decreased work performance, even after controlling for depressive and anxiety disorders. Furthermore, co-occurrence of BPD and affective disorders is common. Therefore, we examined the effect of BPD symptoms on occupational functioning in workers with affective disorders.

**Methods:**

Healthy workers (*n = 287*), workers with current depression/anxiety only (*n = 195*), workers with BPD symptoms only (*n = 54*), and workers with both depression/anxiety and BPD symptoms (*n = 103*) were selected from the Netherlands Study of Depression and Anxiety (NESDA). Both a categorical and dimensional approach were used to cross-sectionally study the effect of BPD symptoms on work performance and absenteeism.

**Results:**

Compared to healthy controls, all symptomatic groups had impaired occupational functioning. Workers with current depression/anxiety had higher long-term absenteeism (OR = 3.59; 95%CI:1.83–7.02) and impaired work performance (OR = 7.81; 95%CI:4.44–13.73), workers with BPD symptoms only had higher impaired work performance (OR = 6.02 95%CI:2.76–13.09), and workers with both depression/anxiety and BPD symptoms had higher long-term absenteeism (OR = 3.66 95%CI:1.69–7.91) and impaired work performance (OR = 10.41 95%CI:5.38–20.15). No difference was found between the (symptomatic) groups. In the dimensional analysis, all associations between BPD symptoms and occupational measures disappeared when depressive symptoms were added. Depressive and BPD symptoms were highly correlated (r = .67).

**Conclusions:**

Our findings confirm that both affective disorders and BPD symptoms are associated with occupational dysfunction. The effect of BPD symptoms however, seems mediated by depressive symptoms. This would suggest that focusing on affective symptoms in occupational health may be effective to improve occupational functioning in persons with BPD.

## Background

Borderline personality disorder (BPD) is a mental illness characterized by instability in interpersonal relationships, self-image, emotion regulation and impulse control [1]. BPD is furthermore associated with suicidal behaviour, severe functional impairment and high rates of comorbid mental disorders [1]. The prevalence of BPD is estimated to vary between 0.5 and 1.4% in the general population [2–6]. In clinical populations prevalence estimates vary between 10% of outpatients to 25% of inpatients [1, 7]. Although BPD symptoms respond to treatment and naturally decrease over time, occupational functioning often remains severely impaired in patients with BPD irrespective of clinical symptom remission [8, 9].

In the Netherlands, a dose-response relationship was found between increasing number of BPD symptoms and unemployment. Of those with 1–2 BPD symptoms 25.6% were unemployed up to 47.4% of those with ≥5 symptoms (the threshold for clinical BPD diagnosis) [4]. However, when examining workers in the general Dutch population, we found that symptoms of BPD were associated with impaired work performance, even after controlling for common mental disorders (CMD) [10]. This demonstrates the negative impact of BPD symptoms on work performance. In workers with BPD, occupational dysfunction is found to be related to relational conflicts with supervisors and co-workers, high sensitivity to criticism, ineffective task strategies and avoidance of certain tasks and procrastination [11–13]. In addition, the BPD symptom-domains impulsivity and affective instability were associated with diminished academic achievement [14].

Decreased work performance and unemployment in BPD lead to considerable societal costs [15, 16]. It has been suggested that the total societal costs related to BPD are largely attributable to productivity loss [17]. Furthermore, unemployment related costs in BPD exceed those in mood and anxiety disorders [18–22] due to a larger employment gap. This suggests that indirect costs of BPD are higher than those for affective disorders. However, only a limited number of studies on occupational functioning take BPD into account. Instead the majority of studies focuses on other, more common mental disorders, such as mood and anxiety disorders [23–26]. BPD and affective disorders however, very often co-occur, emphasising the necessity to investigate how both domains of psychopathology interact in their effects on occupational functioning [4, 22, 27]. Disregarding BPD, may for instance lead to an overestimation of the effects of depression and anxiety.

Therefore, the primary aim of the present study was to examine the association of BPD with absenteeism and work performance, as measures of occupational functioning, in workers with and without affective disorders as assessed in the Netherlands Study of Depression and Anxiety (NESDA). We looked at BPD using both categorical (likely diagnosis) and dimensional (severity of symptoms) levels of case-ness and also considered specific domains of BPD pathology (affective instability, identity problems, negative relationships and self-harm as continuous measures). Given the previously found association between impaired work performance and BPD symptoms in workers from the general population, we hypothesize that impaired work performance in individuals with affective disorders is partly explained by their BPD symptoms.

## Methods

### Study population

For this study we used data of the 6-year follow up assessment from the Netherlands Study of Depression and Anxiety (NESDA). This is a longitudinal, naturalistic cohort study designed to investigate the course and consequences of depressive and anxiety disorders (*n* = 2981) [28]. Participants, aged 18 to 65 years, with a current or past anxiety and/or depressive disorder, and healthy controls were recruited from the community, primary care and specialized mental health care. The presence of depressive or anxiety disorders was determined using the DSM-IV based Composite International Diagnostic Interview (CIDI, version 2.1). Exclusion criteria for the study were: 1) being insufficiently fluent in Dutch, and 2) having been diagnosed with a primary clinical diagnosis of a psychotic disorder, obsessive compulsive disorder, bipolar disorder or severe addiction disorder. For the rationale, objectives and methods of NESDA see Penninx et al. [28]. The NESDA study protocol was approved by the Ethical Review Board of all participating centres (reference no. 2003/183) and all participants provided informed consent. Data of the 6-year assessment (*n* = 2256 (75.7%)) was used for the current study, as this was the wave in which the Personality Assessment Inventory (PAI-BOR) was included in the assessment (*n* = 2143). For the present study, we selected participants with (i) PAI-BOR data, and in line with a previous study, who reported to be (ii) in a paid job of more than 8 h per week [29]. These participants could be in sickness benefits, but individuals performing voluntary work or on maternity leave were excluded, resulting in a total study sample of 637 participants.

### Measures

#### Depression and anxiety diagnoses

For the assessment of DSM-IV diagnoses of depressive and anxiety disorder the CIDI lifetime interview, version 2.1 was used [30]. Current diagnoses (past 6 months) of depressive disorders (major depressive disorder and dysthymic disorder) and anxiety disorders (social phobia, panic disorder (with and without agoraphobia) and generalized anxiety disorder (GAD)) were used. Severity of depressive symptoms (last week) was assessed by means of the 30-item Inventory for Depressive Symptomatology (IDS) questionnaire [31] and used as a continuous variable. Internal consistency of the IDS in NESDA was previously found to be good (Cronbach’s α = 0.91) [32]. Severity of anxiety symptoms (last week) was assessed by means of the 21-item Beck Anxiety Inventory (BAI), [33], also used as a continuous variable. Internal consistency of the BAI in NESDA was previously found to be good (Cronbach’s α = 0.94) [32].

#### Borderline personality disorder symptoms

For the assessment of BPD symptoms, the 24-item self-report Personality Assessment Inventory (PAI-BOR) was used [34]. Internal consistency of the Dutch version of the PAI-BOR is good (Cronbach’s α = 0.81) [35]. The PAI-BOR consists of four subscales, with six items each on four-point rating scales ranging from 0 (false) to 3 (very true). The subscale affective instability (BOR-A α = 0.74) examines the tendency to switch between negative and positive affect, specifically in response to the interpersonal environment. The subscale identity problems (BOR-I α = 0.71) measures the consistency of self-identity. The subscale negative relationships (BOR-N α = 0.63) refers to the propensity of involvement in intense and unstable relationships. The subscale self-harm (BOR-S α = 0.68) examines the tendency of impulsive or self-damaging behaviour.

The Dutch translation of the PAI-BOR was found to discriminate well between those with significant BPD features and those with a relative absence of BPD features [35]. In previous studies, incremental validity was shown for the PAI-BOR in a population sample [36], and concurrent validity was found in assessing patients with SCID-II BPD diagnoses [37]. According to the PAI-BOR manual a total score, based on all subscales, can be calculated (BOR-TOT, α = 0.87). A total score of < 59 reflects an average score, a total score from 60 to 69 reflects an elevated score and a total score of > 70 reflects significant BPD symptoms. A score of significant BPD symptoms in combination with above average scores on the PAI-BOR subscales suggests that a DSM-IV BPD diagnosis is highly likely [27, 34, 35].

#### Occupational functioning

In line with Plaisier et al. [29], occupational functioning was conceptualized in terms of absenteeism and work performance. These were assessed with the Health and Labour Questionnaire Short Form (SF-HLQ) of the TiC-P (Trimbos/iMTA Questionnaire for costs associated with Psychiatric Illnesses) [38]. The TiC-P has been widely used in large population studies and has good validity and reliability [26, 39]. Absenteeism was based on self-report and expressed by the number of weeks absent from work in the last 6 months. This was computed by dividing the number of days absent from work by the number of workdays a person was supposed to work. Absenteeism was not normally distributed, most participants reported not being absent. In line with previous work on absenteeism, it was categorized into three categories: no absenteeism, short-term absenteeism (< 2 weeks in last 6 months) and long-term absenteeism (> 2 weeks in last 6 months) [29, 40, 41]. Also, this cut-off between short-term and long-term absenteeism was used to represent a sensible distinction between short-term absenteeism more likely to be due to common health conditions, such as colds and flu, compared to long-term absenteeism which is more likely due to chronic conditions.

Work performance was based upon two self-report questions of the TiC-P: 1) “On how many days during the last 6 months did you perform paid work, although you were bothered by health problems?”, and 2) “Please rate how well you performed on the days you went to work even though you were suffering from health problems” on a 10-point scale (ranging from 0.0 = maximally inefficient to 1.0 = efficient as usual). Work performance was not normally distributed. In line with previous research, work performance was computed based on the following formula [29, 42, 43]:

$$ work\ performance=\frac{no. days\ hindered\ast \left(1- efficiency\right)\ast work\ hours\  per\  day}{no. work\ hours\  per\  week} $$

A higher outcome indicates more decreased work performance. This variable ranged from 0 to 39.8 and was not normally distributed. Therefore, in line with previous research, work performance was categorized in no impairment (0 days), reduced work performance (> 0–1.68 days), and impaired work performance (> 1.68 days) [29, 42, 43]. Again, the cut-off represents a sensible distinction between reduced and impaired work performance.

#### Covariates

In line with previous research on occupational functioning [29] putative confounding variables were gender, age, education (in years), the number of working hours per week, and the number of ever experienced self-reported somatic conditions consisting of the sum of heart diseases, diabetes, stroke, arthritis, cancer, hypertension, intestinal problems, liver disease, epilepsy, chronic lung problems, allergy and injuries.

### Statistical analyses

To examine absenteeism and work performance related to BPD we used two approaches, a categorical and a dimensional approach. For the categorical approach, we first defined likely BPD diagnosis based on the PAI-BOR (significant BPD symptoms and above average scores on all subscales). We then composed 4 groups: (1) Healthy controls (no lifetime depression/anxiety and no likely BPD diagnosis), (2) Current depression/anxiety and no likely BPD diagnosis, (3) likely BPD diagnosis without current depression/anxiety, and (4) Current depression/anxiety and likely BPD diagnosis. Differences in socio-demographics and work-related outcomes between the 4 groups were examined using analyses of variance (ANOVA) for continuous variables, chi-square statistics for categorical variables, and Kruskal-Wallis for non-parametric variables. For the dimensional approach, we used the PAI-BOR total score.

Multinomial logistic regression was performed to test the associations between the diagnostic group (categorical predictor) and absenteeism and work performance (outcomes), while additionally controlling for covariates (Model 1). Also, absence ratio based on the number of absent workweeks was added as a covariate in the analyses of work performance, because for those reporting absence, fewer days had to be left out to assess actual work performance [29]. Odds ratios and 95% confidence intervals were calculated for short-term and long-term absenteeism compared to no absenteeism, and for decreased and impaired work performance compared to no change in work performance.

The analyses were repeated with BPD symptoms (dimensional) as a predictor. In these analyses, the associations with absenteeism and work performance with the four PAI-BOR domains (affective instability, identity problems, negative relationships and self-harm) were also analysed. Next, we extended the models including severity of depression and anxiety to see if effects of BPD symptoms were independent of depression and anxiety (Model 2). Severity of anxiety symptoms (BAI) was highly correlated with severity of depressive symptoms (IDS) (r = .76), and was therefore omitted from the analyses. Data was analysed using SPSS 22.0 and statistical significance was set at *p* ≤ .05.

## Results

### Sample description

Of the 637 workers included, 287 (45.0%) had no current depressive/anxiety disorder or likely BPD diagnosis, 195 (30.5%) had current depressive/anxiety disorder and no likely BPD diagnosis, 54 (8.4%) had likely BPD diagnosis without current depressive/anxiety disorder, and 103 (16.1%) workers had both current depressive/anxiety disorder and likely BPD diagnosis. Education in years, number of working hours and number of somatic diseases differed significantly across groups (Table 1).

**Table 1:** Demographics, health characteristics and work outcomes in workers (*n* = 637) by diagnostic group

	Healthy controls (*n* = 287)	Current depressive/anxiety disorder and no likely BPD diagnosis (*n* = 195)	Likely BPD diagnosis without current depressive/anxiety disorder (*n* = 54)	Current depressive/anxiety disorder + likely BPD diagnosis (*n* = 103)	*p*-value^a^
Sex, % female	57.5	67.2	59.3	62.1	0.19
Age, mean in years (SD)	43.8 (12.6)	44.5 (10.4)	42.7 (11.2)	43.3 (10.9)	0.71
Education, mean in years (SD)	14.1 (3.1)	13.3 (3.3)	12.3 (3.3)	12.0 (3.4)	**< 0.001**
Working hours, mean no. hours per week (SD)	32.8 (9.4)	30.4 (10.4)	31.8 (9.0)	31.2 (9.5)	**0.048**
Number of somatic diseases, median (IQR)	0.0 (0.0–1.0)	0.0 (0.0–1.0)	0.0 (0.0–1.0)	1.0 (0.0–1.0)	**0.007**
Work absenteeism, median (IQR)	0.0 (0.0–1.0)	1.0 (0.0–1.0)	0.0 (0.0–1.0)	0.0 (0.0–1.0)	
Work absenteeism (%)					**< 0.001**
No absence	67.9	49.7	53.7	52.4	
Short-term absence	26.5	32.8	35.2	28.2	
Long-term absence	5.6	17.4	11.1	19.4	
Work performance rate, median (IQR)	0.0 (0.0–0.0)	1.0 (0.0–2.0)	1.0 (0.0–2.0)	1.0 (0.0–2.0)	
Work performance rate (%)					**< 0.001**
No changed work performance	76.3	36.4	46.3	32.0	
Reduced work performance	15.7	29.7	22.2	26.2	
Impaired work performance	8.0	33.8	31.5	41.7	
Severity of depressive symptoms (IDS scores), mean (SD)	4.8 (4.0)	20.0 (9.8)	23.0 (8.2)	27.5 (10.9)	**< 0.001**
Severity of anxiety symptoms (BAI scores), mean (SD)	2.4 (3.0)	12.1 (8.2)	12.5 (7.8)	16.8 (10.9)	**< 0.001**
DSM-IV BPD diagnosis is highly likely (%)	0.0	0.0	37.0	38.8	**< 0.001**

^a^ Based on ANOVA for continuous, chi-square for dichotomous and Kruskal-Wallis for non-parametric variables

Significant *p-*values highlighted in bold

### Relation between psychopathology and absenteeism and work performance

In the categorical approach, absenteeism and work performance differed significantly across groups. Table 1 shows that the lowest rates of absenteeism and impaired work performance were found in the control group, followed by the likely BPD diagnosis without current depressive/anxiety disorder group. The current depressive/anxiety disorder and no likely BPD diagnosis and the group with both current depressive/anxiety disorder and likely BPD diagnosis showed the highest rates on absenteeism and impaired work performance. There were no differences between the (symptomatic) groups.

The adjusted associations between absenteeism and work performance in the three subgroups compared to the healthy control group are shown in Table 2. The depression & anxiety only group was significantly associated with both short-term (OR = 1.76; 95%CI:1.15–2.69) and long-term absenteeism (OR = 3.59; 95%CI:1.83–7.02). The group with depression & anxiety and BPD diagnosis likely was significantly associated with long-term absenteeism (OR = 3.66; 95%CI:1.69–7.91). Although the OR for especially short-term absenteeism was not much different from the ORs in other groups, the BPD only group was not significantly associated with absenteeism (short-term absenteeism OR = 1.80; 95%CI:0.93–3.47, and long-term absenteeism OR = 2.04; 95%CI:0.71–5.87). In post-hoc analysis comparing the BPD only group with the other case groups, no significant differences were observed. With respect to work performance, the depression & anxiety group with likely BPD diagnosis (OR = 10.41; 95%CI:5.38–20.15), the depression & anxiety only group (OR = 7.81; 95%CI:4.44–13.73), and the group with likely BPD diagnosis only (OR = 6.02; 95%CI:2.76–13.09) were significantly associated with impaired work performance. Again, comparison of the BDP with other case groups did not reveal differences between the groups.

**Table 2:** Multinomial logistic regression between group and absenteeism and work performance in workers (*n* = 637)

Absenteeism	Short-term absenteeism^b^		Long-term absenteeism^b^	
	OR (95% CI)	*p*	OR (95% CI)	*p*
D/A only^a^	1.76 (1.15–2.69)	**0.01**	3.59 (1.83–7.02)	**< 0.001**
BPD symptoms only^a^	1.80 (0.93–3.47)	0.08	2.04 (0.71–5.87)	0.19
D/A + BPD symptoms^a^	1.51 (0.87–2.61)	0.14	3.66 (1.69–7.91)	**0.001**
Work performance	Reduced work performance^c^		Impaired work performance^c^	
	OR (95% CI)	*p*	OR (95% CI)	*p*
D/A only^a^	3.95 (2.42–6.42)	**< 0.001**	7.81 (4.44–13.73)	**< 0.001**
BPD symptoms only^a^	2.29 (1.05–4.98)	**0.04**	6.02 (2.76–13.09)	**< 0.001**
D/A + BPD symptoms^a^	3.83 (2.05–7.17)	**< 0.001**	10.41 (5.38–20.15)	**< 0.001**

^a^ Reference category: Control group

^b^ Reference category: No absenteeism

^c^ Reference category: No impaired work performance

Adjusted for covariates: sex, age, education, number of somatic diseases and working hours; and additionally absence in the model for reduced and impaired work performance

Significant *p-*values highlighted in bold

Concerning the dimensional approach, Table 3 shows the associations between the dimensional BPD score and BPD domains with absenteeism and work performance. BPD symptoms were significantly associated with long-term absenteeism (OR = 1.03; 95%CI:1.00–1.05) in model 1. The BPD domain affective instability was associated with both short-term (OR = 1.06; 95%CI:1.01–1.10), and long-term absenteeism (OR = 1.08; 95%CI:1.01–1.15). However, when adding severity of depression to model 2, the associations between BPD symptoms and affective instability with absenteeism disappeared. In this model, only severity of depression was associated with long-term absenteeism (OR = 1.05; 95%CI:1.02–1.07). BPD symptoms, affective instability, identity problems and negative relationships were all significantly associated with both reduced and impaired work performance in model 1. In addition, self-harm was significantly associated with impaired work performance. However, again in model 2 when adding severity of depression, all significant associations disappeared except for severity of depression with reduced and impaired work performance, and affective instability with reduced work performance (OR = 1.08; 95%CI:1.00–1.16).

**Table 3:** Multinomial logistic regression between borderline personality symptoms (continuous) and absenteeism and work performance in workers (*n* = 637)

Absenteeism	Short-term absenteeism^a^		Long-term absenteeism^a^	
	OR (95% CI)	*P*	OR (95% CI)	*P*
Model 1				
Borderline personality disorder symptoms	1.02 (1.00–1.03)	0.052	1.03 (1.00–1.05)	**0.03**
Affective Instability	1.06 (1.01–1.10)	**0.02**	1.08 (1.01–1.15)	**0.02**
Identity Problems	1.03 (0.99–1.08)	0.18	1.06 (0.99–1.14)	0.07
Negative Relationships	1.05 (0.99–1.10)	0.09	1.05 (0.97–1.12)	0.22
Self-harm	1.03 (0.96–1.10)	0.49	1.08 (0.99–1.19)	0.09
Model 2				
Borderline personality disorder symptoms	1.00 (0.98–1.02)	0.94	0.99 (0.96–1.03)	0.65
Severity of depression	1.02 (0.98–1.04)	0.08	1.05 (1.02–1.07)	**0.003**
Affective Instability	1.02 (0.96–1.09)	0.51	0.99 (0.91–1.09)	0.88
Identity Problems	0.97 (0.91–1.04)	0.42	0.95 (0.86–1.04)	0.27
Negative Relationships	1.01 (0.95–1.07)	0.72	0.97 (0.89–1.06)	0.52
Self-harm	1.00 (0.92–1.07)	0.91	1.03 (0.94–1.14)	0.53
Work performance	Reduced work performance^b^		Impaired work performance^b^	
	OR (95% CI)	*P*	OR (95% CI)	*P*
Model 1				
Borderline personality disorder symptoms	1.04 (1.02–1.06)	**< 0.001**	1.07 (1.05–1.09)	**< 0.001**
Affective Instability	1.15 (1.09–1.21)	**< 0.001**	1.21 (1.14–1.28)	**< 0.001**
Identity Problems	1.10 (1.04–1.16)	**0.001**	1.22 (1.16–1.30)	**< 0.001**
Negative Relationships	1.08 (1.02–1.15)	**0.007**	1.16 (1.09–1.23)	**< 0.001**
Self-harm	1.04 (0.96–1.13)	0.35	1.11 (1.03–1.20)	**0.007**
Model 2				
Borderline personality disorder symptoms	1.00 (0.98–1.03)	0.82	1.01 (0.98–1.03)	0.62
Severity of depression	1.05 (1.02–1.08)	**< 0.001**	1.09 (1.06–1.12)	**< 0.001**
Affective Instability	1.08 (1.00–1.16)	**0.04**	1.03 (0.95–1.11)	0.38
Identity Problems	0.98 (0.90–1.06)	0.54	1.02 (0.95–1.11)	0.58
Negative Relationships	0.99 (0.93–1.06)	0.86	1.01 (0.94–1.08)	0.89
Self-harm	0.97 (0.89–1.06)	0.46	0.99 (0.91–1.08)	0.83

^a^ Reference category: No absenteeism

^b^ Reference category: No impaired work performance

Model 1: Adjusted for covariates: sex, age, education, number of somatic diseases and working hours and absence in the model for reduced and impaired work performance

Model 2: Adjusted for all covariates in Model 1 and severity of depression

Significant *p-*values highlighted in bold

Because adding severity of depression to the dimensional model led to the association between BPD symptoms and absenteeism (and to some extent work performance) becoming non-significant, we calculated Pearson correlations. This revealed modest to strong correlations between depressive symptoms and BPD symptoms total score, and with all subscales of the PAI-BOR (affective instability, identity problems, negative relationships, and self-harm) (*p* = < 0.001) (Table 4). Depressive symptoms were strongly associated with BPD symptoms (*r* = .67), affective instability (*r* = .61), and identity problems (*r* = .67).

**Table 4:** Correlations among severity of depressive symptoms, borderline personality disorder symptoms and borderline personality disorder domain variables

	1	2	3	4	5	6	7
1 Depressive symptoms		.67*	.61*	.67*	.48*	.30*	.59*
2 BPD symptoms			.85*	.83*	.82*	.61*	.57*
3 Affective Instability				.64*	.58*	.36*	.58*
4 Identity Problems					.57*	.32*	.50*
5 Negative Relationships						.38*	.47*
6 Self-Harm							.27*
7 Grouping variable							

N = 637, * *p* < 0.001

*BPD* Borderline personality disorder

## Discussion

To our knowledge, this was the first study examining the independent effect of BPD (likely) diagnosis and symptom domains on absenteeism and work performance in individuals with (and without) current depression and anxiety. Both BPD and depression and anxiety were associated with impaired occupational functioning, but effects of BPD symptoms in absenteeism and impaired work performance seemed to be mediated by depression/anxiety. The different patient groups (current depression & anxiety with and without likely BPD diagnosis and the likely BPD diagnosis only group) predominantly exhibited reduced and impaired work performance, and to a lesser extent absenteeism compared to healthy controls. BPD symptoms as a dimensional measure were associated with long-term absenteeism and both reduced and impaired work performance. However, these associations disappeared when adding severity of depressive symptoms to the models.

The present study confirms previously found impaired work performance in workers with psychopathology [23, 29]. This may be explained by the fact that a large part of the present sample consisted of individuals clinically diagnosed with affective disorders. Furthermore, although BPD symptoms were measured at the (non-clinical) symptomatic level, comorbidity of BPD (symptoms) and affective disorders increased impaired work performance as previously reported [22]. This coincides with previous studies demonstrating that severity of psychiatric disorders increased impaired work performance [29, 44]. Although we did not find significant effects for the BPD only group with both short- and long-term absenteeism, effect sizes were comparable to the significant effect sizes in the depressive/anxiety group with and without likely BPD diagnosis, and no differences were observed when comparing the BPD only group with the other case groups. The BPD only group was relatively small, which may explain the wider confidence intervals for the BPD only group.

Contrary to previous findings [10, 45, 46], the association with BPD symptoms disappeared when controlling for depressive symptoms. However, the correlation we found between depressive symptoms, BPD symptoms, and the different BPD domains contributes to the literature by showing that comorbidity between depressive disorders and BPD is high and that symptoms overlap [22, 27]. One of the shared vulnerabilities in individuals with comorbid depression, anxiety, and BPD symptoms is the personality trait neuroticism [47, 48]. Neuroticism has been shown to be associated with impaired work functioning [49–51]. Neuroticism is characterized by being easily upset, maladjusted, and not being calm [52], and it has been previously suggested that improving problems solving skills in workers with high neuroticism may diminish their vulnerability to stress [49]. Furthermore, costs of neuroticism are found to exceed those of common mental disorders and are to a large extent related to production losses stemming from absenteeism [51]. Still, apart from the BPD domains studied, other disorder-specific traits remain which were not examined in the present study, such as impulsivity and hostility in BPD, pessimism in depression, and perfectionism in anxiety [27, 49].

To our knowledge and in line with our hypothesis, this study was the first to show that the BPD domains affective instability, identity problems and negative relationships were associated with both reduced and impaired work performance, and self-harm with impaired work performance. Affective instability and impaired work performance were previously found to be related to diminished academic achievement [14]. However, apart from the association between affective instability and reduced work performance, all associations with the separate BPD domains disappeared after adjusting for severity of depressive symptoms.

### Limitations

Although the study provided the unique opportunity to examine and compare the association between BPD symptoms, depressive and anxiety disorders with both absenteeism and work performance, there are also limitations. First, the present findings are based on cross-sectional analyses. Consequently, it is not possible to draw any conclusions about causality. Longitudinal studies are needed to assess long-term consequences of diagnosis on occupational functioning and tease out temporal sequences of perceived shared vulnerabilities between BPD symptoms and affective disorders. Second, BPD symptoms in this sample were not examined by means of a clinical interview but by means of a self-report questionnaire. BPD is often under-detected [53–55] and it is therefore conceivable that BPD symptoms were under-recognized in this sample. Misclassification of BPD symptoms might have led to an underestimation of the contribution of BPD symptoms to depressive/anxiety disorders with respect to work performance. Third, absenteeism and work performance were based on self-report. This self-report might not correspond with employer payroll records. However, previously high correspondence was found between self-report and employer payroll records [56]. In addition, the reasons for absenteeism and reduced work performance were not assessed and may be biased due to current diagnosis or symptoms. Fourth, adverse working conditions such as high job demands, low decision latitude, low skill discretion, low social support and low job security are important predictors of occupational dysfunction in both healthy and psychiatric workers [49, 57, 58] and were not assessed here. Also, type of industry or job and increased pressure of higher labour flexibility by reforming labour market regulation and working arrangements appeal to workers’ performance capabilities [58]. Other factors of performance or occupational functioning such as job position, information on resignation, dismissal or demotion were unfortunately also not assessed. Fifth, given the objectives of NESDA, the sample is not representative for workers in the general population, workers with BPD, or the entire BPD population. However, NESDA is representative of a population with depressive and anxiety disorders, which is a strength given the aims of our study. Sixth, the NESDA study was originally set up to study course and consequences of depression and anxiety, but not specifically to evaluate the role of BPD symptomatology. Sample sizes between groups differed, however, effect sizes were comparable. Because the original study was not specifically set up to examine work performance and absenteeism in workers with BPD symptoms, the group of workers with BPD symptoms was smaller as compared to the other groups, it is therefore conceivable that a type II error has occurred. Therefore, the results should be interpreted with caution. In general, future cohort studies should include samples of individuals with clinically diagnosed BPD, with efficient sample sizes, with follow-up assessments on measures of absenteeism and work performance, and investigate the role of working conditions on work performance of workers with BPD.

### Clinical implications

This study offers insight into the need of a better recognition and support of (any psychiatric) symptoms to reduce impaired work performance. It is known that individuals with psychiatric disorders have difficulty discussing their symptoms and vulnerabilities due to a fear of stigmatization. Therefore, overcoming difficulties in and barriers to work should be integrated in psychiatric treatment as maintaining employment is most likely positively contributing to health and mood. For example, reducing absenteeism could be a clear goal in the treatment plan. Future longitudinal studies should examine the question to what extent mood, anxiety, BPD symptoms, and shared vulnerabilities affect work performance more thoroughly. A more concise examination of which symptoms affect occupational functioning will provide new strategies to support and improve performance in workers with these mental health vulnerabilities and could be incorporated as goals for improvement in a treatment plan.

## Conclusions

The present study confirms that both depressive and anxiety disorders and BPD symptoms are important factors for absenteeism and impaired work performance, and highlights the need to support these individuals in the work process. An important lead for further investigation is that, in the present study, occupational dysfunction in BPD symptoms was mediated by affective symptoms. This might suggest that work impairment in BPD is explained by affective symptoms which could be used to inform clear treatment goals to improve functioning. Despite the limitation of only having access to cross-sectional data, the present findings suggest that it is important to study mood, anxiety and BPD symptoms in relation to occupational functioning, together with the contribution of negative working conditions as these may provide important implications for strategies to improve occupational functioning in these workers. Therefore, future studies should examine mental health vulnerabilities together with working conditions in close collaboration with mental health and occupational health professionals and stakeholders from the workplace in order to inform strategies aiming to improve occupational functioning.

## Data Availability

The datasets generated and/or analysed during the current study are not publicly available due to participant privacy and the consent provided.

## Abbreviations

ANOVA: Analyses of variance
BAI: Beck anxiety inventory
BPD: Borderline personality disorder
CI: Confidence interval
CIDI: Composite international diagnostic interview
CMD: Common mental disorder
GAD: Generalized anxiety disorder
IDS: Inventory for depressive symptomatology questionnaire
NESDA: Netherlands study of depression and anxiety
OR: Odds ratio
PAI-BOR: Personality assessment inventory
SF-HLQ: Health and labour questionnaire short form
TiC-P: Trimbos/iMTA questionnaire for costs associated with psychiatric illnesse
